# Orthosteric and/or Allosteric Binding of α-Conotoxins to Nicotinic Acetylcholine Receptors and Their Models

**DOI:** 10.3390/md16120460

**Published:** 2018-11-22

**Authors:** Elena V. Kryukova, Igor A. Ivanov, Dmitry S. Lebedev, Ekaterina N. Spirova, Natalia S. Egorova, Marios Zouridakis, Igor E. Kasheverov, Socrates J. Tzartos, Victor I. Tsetlin

**Affiliations:** 1 Shemyakin-Ovchinnikov Institute of Bioorganic Chemistry, Russian Academy of Sciences, Miklukho-Maklaya Street, 16/10, 117997 Moscow, Russia; evkr@mail.ru (E.V.K.); chai.mail0@gmail.com (I.A.I.); dmitry-1@bk.ru (D.S.L.); katya_spirova@mail.ru (E.N.S.); natalyegorov@yandex.ru (N.S.E.); shak_ever@yahoo.com (I.E.K.); 2 Department of Neurobiology, Hellenic Pasteur Institute, 127, Vas. Sofias ave., Athens 115 21, Greece; marzouri@gmail.com (M.Z.); stzartos@gmail.com (S.J.T.); 3 Institute of Molecular Medicine, Sechenov First Moscow State Medical University, Trubetskaya Street 8, bld. 2, 119991 Moscow, Russia; 4 PhysBio of MEPhI, Kashirskoye Ave., 31, 115409 Moscow, Russia

**Keywords:** conotoxins, nicotinic acetylcholine receptors, ligand-binding domain, acetylcholine-binding protein, binding site, radioligand analysis

## Abstract

α-Conotoxins from *Conus* snails are capable of distinguishing muscle and neuronal nicotinic acetylcholine receptors (nAChRs). α-Conotoxin RgIA and αO-conotoxin GeXIVA, blocking neuronal α9α10 nAChR, are potential analgesics. Typically, α-conotoxins bind to the orthosteric sites for agonists/competitive antagonists, but αO-conotoxin GeXIVA was proposed to attach allosterically, judging by electrophysiological experiments on α9α10 nAChR. We decided to verify this conclusion by radioligand analysis in competition with α-bungarotoxin (αBgt) on the ligand-binding domain of the nAChR α9 subunit (α9 LBD), where, from the X-ray analysis, αBgt binds at the orthosteric site. A competition with αBgt was registered for GeXIVA and RgIA, IC_50_ values being in the micromolar range. However, high nonspecific binding of conotoxins (detected with their radioiodinated derivatives) to His_6_-resin attaching α9 LBD did not allow us to accurately measure IC_50_s. However, IC_50_s were measured for binding to *Aplysia californica* AChBP: the RgIA globular isomer, known to be active against α9α10 nAChR, was more efficient than the ribbon one, whereas all three GeXIVA isomers had similar potencies at low µM. Thus, radioligand analysis indicated that both conotoxins can attach to the orthosteric sites in these nAChR models, which should be taken into account in the design of analgesics on the basis of these conotoxins.

## 1. Introduction

**Figure 1:**
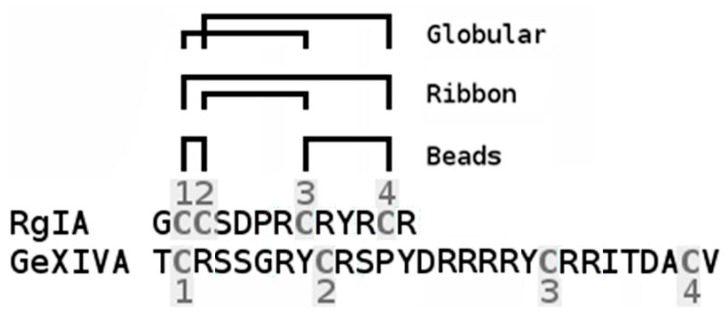
Amino acid sequences of α-conotoxin RgIA and αO-conotoxin GeXIVA with scheme of formation of their three isomers (globular, ribbon and beads) by forming disulfides between 4 cysteine residues (shaded grey and numbered).

Protein neurotoxins from snake venoms played a key role first in the isolation of nAChRs, and then in their structural and functional studies (see reviews [1,2,3,4,5,6]). At present, muscle-type and neuronal nAChRs are known, and also the so-called "non-neuronal" nAChRs consisting of the same subunits as neuronal ones. Non-neuronal nAChRs are found not in the brain, but in other tissues, in particular in immune system cells [7,8,9]. Snake venom neurotoxins interact mainly with the muscle nAChRs, as well as with the receptors built from α7 or α9α10 subunits [2]. α-Conotoxins, neurotoxic peptides from venomous Conus sea mollusks, appeared in the nAChRs research later, but it is the variety of natural α-conotoxins and their numerous synthetic analogues that allows us today to not only accurately discriminate between the muscle and neuronal nAChRs, but also to identify of various subtypes of the latter [2,10,11,12,13]. In particular, α-conotoxins RgIA (Figure 1) and Vc1.1 (containing 13 and 16 amino acid residues, respectively, and 2 disulfides) have a high selectivity for α9α10 nAChRs, and are considered as promising analgesics [14,15,16,17]. Recently, a new αO-conotoxin GeXIVA with different cysteine framework (Figure 1) has been discovered, which also blocked α9α10 nAChR, and was effective against neuropathic pain [18,19,20]. Naturally-occurring α-conotoxins, blocking muscle and α7 nAChRs, compete with radioactive αBgt for binding to these receptors or to the acetylcholine-binding proteins (AChBPs), thus indicating their interaction with the orthosteric sites [11,21,22,23,24]. Moreover, binding to the orthosteric sites of α-conotoxins was observed by X-ray analysis of their complexes with AChBPs [11,25,26,27,28]. On the other hand, electrophysiological studies on α9α10 nAChRs expressed in *Xenopus* oocytes indicated that, in contrast to "traditional" α-conotoxins, binding of αO-conotoxin GeXIVA (Figure 1) occurs outside the orthosteric site [19]. Given that recently a joint application of electrophysiological methods and radioligand analysis revealed that the interaction of the proteins of the Ly6 family (having the same three-finger structure as snake venom α-neurotoxins) with nAChRs occurs in the orthosteric or allosteric regions, depending on the type of protein and the nAChR subtype [29,30,31,32], in the present work we planned to use radioligand analysis to get more information on the αO-conotoxin GeXIVA binding mode. A similar combination of the above-mentioned methods allowed us to devise a detailed description of the binding modes for novel α-conotoxin PnIA analogues having a high affinity for α7 nAChR [33]. Thus, in the present work, the binding assays were performed using the ligand-binding domain (LBD) of the human α9 subunit described in [34] and the *Aplysia californica* AChBP. 

## 2. Results and Discussion 

### 2.1. Synthesis and Сharacterization of α-Сonotoxin RgIA and αO-conotoxin GeXIVA and Their Iodinated Derivatives 

**Figure 2:**
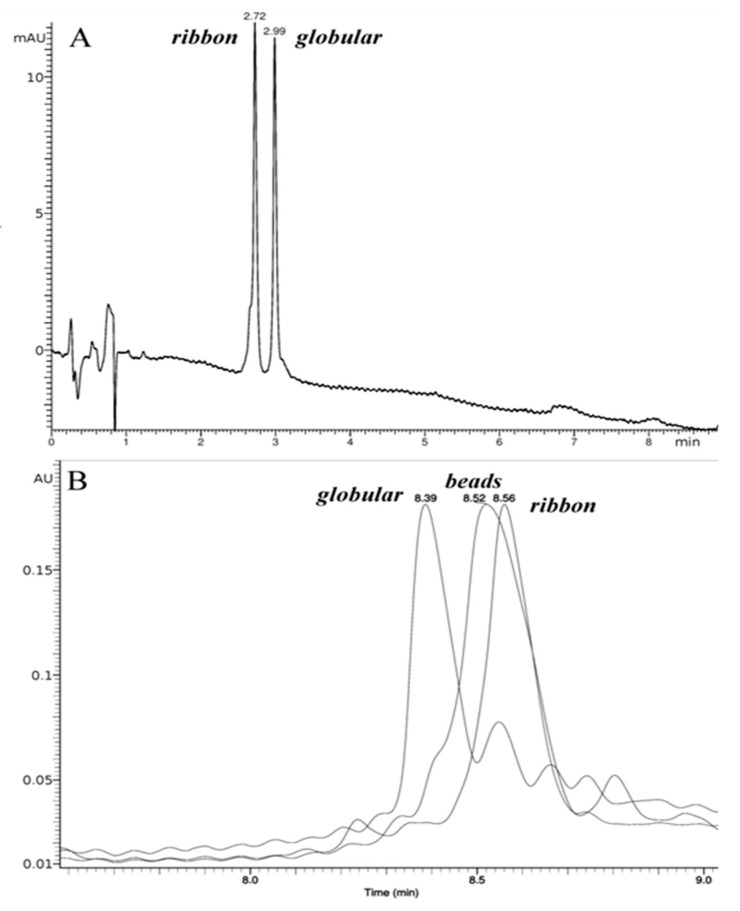
HPLC profiles for synthetic peptides used in this study. (**A**) Co-elution of purified ribbon and globular RgIA isomers in acetonitrile gradient; retention times are shown over respective peaks. (**B**) Superposition of the RP HPLC profiles of analytical chromatograms for purified globular, beads and ribbon GeXIVA isomers; retention times are shown over respective peaks.

Synthesis of linear peptides corresponding to the amino acid sequences of α-conotoxin RgIA and αO-conotoxin GeXIVA was performed by standard methods of solid-phase peptide synthesis. In the case of α-conotoxin RgIA, the strategy of spontaneous oxidation of cysteine sulfhydryl groups was as follows. Two isomers obtained in a 1:2 ratio (ribbon to globular) were separated by reverse-phase (RP) HPLC. Their order of elution (Figure 2A; globular isomer was eluted at higher acetonitrile concentration) agrees with that of isomers separately synthesized with orthogonal protection of Cys pairs, in accordance with the earlier publication [35]. 

The synthesis of αO-conotoxin GeXIVA isomers was, in general, performed as described in [19], using an oxidation strategy with orthogonal protecting groups. For the globular isomer, we also applied the method of closure of the first disulfide bond using the polymer immobilized Ellman’s reagent (CLEAR-OX) [36] (see Materials and Methods), which gave a high yield of folded conotoxin from the linear precursor. The obtained isomers (globular, ribbon, beads) were purified by preparative HPLC with a C5 phase, which showed a better selectivity between the target conotoxin and by-products. The order of elution of the αO-conotoxin GeXIVA isomers (Figure 2B) agrees with the original publication [19]. 

**Figure 3:**
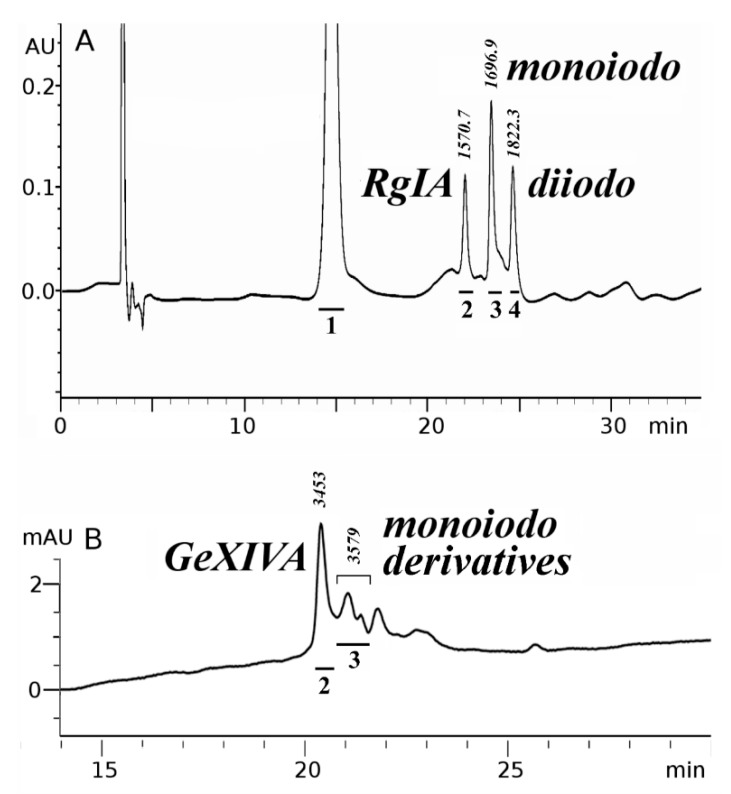
RP HPLC profiles in acetonitrile gradient for the products of the [^127^I]iodination reaction of α-conotoxin RgIA globular isomer (**A**) and αO-conotoxin GeXIVA beads isomer (**B**). The peak of oxidizer (chloramine T, peak 1), non-modified conotoxins (peaks 2), mono- (peaks 3) and di-iodinated derivatives (peak 4) are marked with bars. The numbers above the peaks indicate the corresponding measured molecular masses (Da) obtained by MALDI-TOF mass-spectrometry (**A**) or electrospray HPLC-MS (**B**).

Introduction of iodine atoms (either as nonradioactive isotope-127 or radioactive isotope-125) into RgIA globular isomer or GeXIVA ribbon and beads isomers was performed by chloramine T method which provides highly efficient incorporation of one or two iodine atoms into tyrosine amino acid residues. The presence of only one tyrosine residue (Tyr10) in the RgIA molecule results in the formation of mono- and di-iodinated derivatives, which are easily separated by RP HPLC (Figure 3A). On the other hand, three tyrosine residues in GeXIVA (Tyr8, 13, 19) gave a set of poorly-separated mono-iodinated derivatives (both in the case of ribbon and beads isomers), even under the condition of iodine deficiency in relation to the peptide (see profile for beads isomer in Figure 3B). The structures of all [^127^I]-derivatives of RgIA or GeXIVA were confirmed by MALDI-TOF mass-spectrometry or electrospray HPLC-MS, respectively. In all cases, the found masses (see Figure 3) were in the good agreement with calculated ones (1570.8, 1696.7 and 1822.6 Da for RgIA, [^127^I]-RgIA and [^127^I]_2_-RgIA; 3452 and 3578 Da for GeXIVA and [^127^I]-GeXIVA). 

### 2.2. Analysis of Interaction of Conotoxins with the nAChRs, α9 LBD and A. californica AChBP via Competition with Radioiodinated α-Bungarotoxin

**Figure 4:**
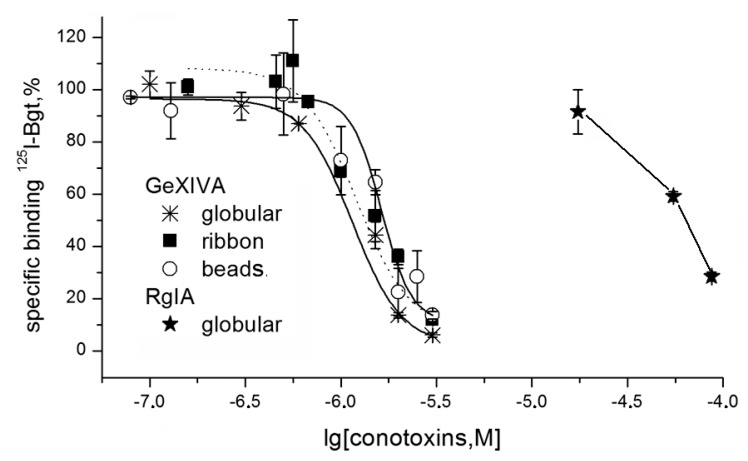
Competition of conotoxins (RgIA globular and three GeXIVA isomers) with [^125^I]-labeled αBgt for binding to α9 LBD. The IC_50_ values (mean ± SEM) for GeXIVA isomers were 1.140 ± 0.012 μM (globular), 1.20 ± 0.05 μM (ribbon, dotted line) and 1.63 ± 0.02 μM (beads), as well as > 60 μM for RgIA globular isomer.

At first, the activity of de-glycosylated α9 LBD [34] was studied by the saturation binding with [^125^I]-labeled αBgt. The dissociation constant was measured in low nanomolar range (data not shown), similarly to data in the original publication [34]. In the competitive radioligand assay, all three isomers of αO-conotoxin GeXIVA could displace [^125^I]-labeled αBgt with similar potencies, but for the globular isomer RgIA, the displacement was notably less efficient (Figure 4). While for αBgt, there is a good correlation between the affinities for the whole-size human α9 nAChR and human α9 LBD [34,37], for both conotoxins, the inhibitory activity towards human α9 LBD was in the micromolar range. It is worthy of note that they both inhibit functionally-active human α9α10 nAChR with the nanomolar constants 494 nM for RgIA globular isomer and 20–116 nM for different GeXIVA isomers [20,38]. It should be kept in mind that the RgIA affinity towards α9 nAChR is strongly species-specific, being 300-fold higher for rat α9α10 nAChR than for the human one [38], and that in the RgIA binding to this receptor, the major contribution is from the α10 subunit surface [14]. These factors may be the reasons for the lower conotoxin affinity towards α9 LBD observed in the competition assay with αBgt. We suspected that this might also be due to a high nonspecific binding of both conotoxins to the Ni^2+^-agarose-beads used to immobilize the His-tagged form of α9 LBD in these radioligand assays. Therefore, this interaction could considerably decrease the amount of toxins available for binding with α9 LBD in solution. We decided to check such a potential binding using radioiodinated RgIA and GeXIVA derivatives. 

**Figure 5:**
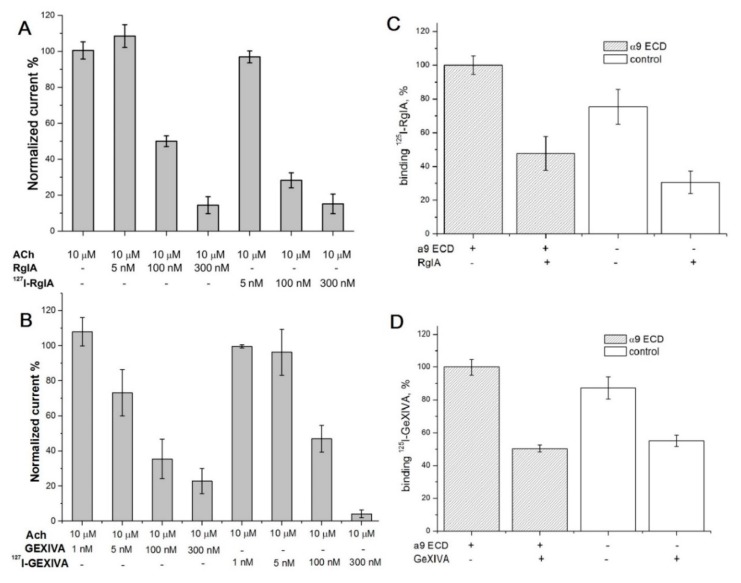
Inhibition of acetylcholine (10 μM)-evoked currents (**A**) through rat α9α10 nAChR by globular isomer of RgIA or its [^127^I]-derivative (5, 100, 300 nM, *n* = 3) and (**B**) through human α9α10 nAChR by ribbon isomer GeXIVA or its [^127^I]-derivative (1, 5, 100, 300 nM, *n* = 2). The bar graph data are presented as mean ± SEM. (**C**) and (**D**) Binding of the radioiodinated conotoxins RgIA globular isomer and GeXIVA beads isomer, respectively, with Ni^2+^-agarose resin. Binding in the presence (dense pattern bars) or absence (open bars) of α9 ECD is shown. α9 LBD binding with the radioiodinated conotoxins on the Ni^2+^-NTA-agarose is regarded as 100%. Each bar is the mean  ±  SEM value of two measurements for each concentration in two (RgIA) or three (GeXIVA) independent experiments.

Firstly, the activity of their non-radioactive [^127^I]iodinated forms (see Figure 3) was checked on the α9α10 nAChR expressed in the *Xenopus* oocytes, and found not to differ significantly—if not being improved—from the activity of the initial conotoxins, as shown in Figure 5A,B for globular [^127^I]-RgIA and ribbon [^127^I]-GeXIVA derivatives, respectively. On these analogs we demonstrated no differences between iodinated and original conotoxins regarding functional tests on the α9α10 nAChR.

Thus, the respective radioiodinated [^125^I]-labeled conotoxin derivatives could be an appropriate tool for analyzing binding to the α9 LBD. However, Figure 5C,D show that both radioactive conotoxins bind not only to the α9 domain, but almost with the same efficiency to the Ni^2+^-agarose-beads. Moreover, a 200-fold molar excess of unlabeled conotoxins displaced the respective radiolabeled derivatives from the resin, regardless of the presence of α9 LBD, indicating that both iodinated and initial RgIA and GeXIVA interact with Ni^2+^-NTA-agarose as well. These data demonstrate that both for RgIA, which is known to bind at the orthosteric site of α9α10 nAChR, and for GeXIVA at the α9 LBD, at least in part, the orthosteric binding may take place, but we cannot reliably estimate its real affinity. 

In general, the results of the α9 LBD study demonstrate that both conotoxins RgIA and GeXIVA bind at its orthosteric site (Figure 4). However, their non-specific interaction with Ni^2+^-agarose resin (Figure 5C,D) does not allow us to draw sound conclusions on their affinities towards α9 LBD by competition assays with [^125^I]-αBgt. 

Therefore, it seemed interesting to investigate how GeXIVA and RgIA interact with the acetylcholine-binding protein (AChBP), which is an excellent model not only for the all types of nAChRs, but for the whole family of Cys-loop receptors [39,40,41]. According to biochemical and X-ray data, AChBP binds neurotoxins and α-conotoxins in the classical orthosteric site, namely, the binding site for agonists and competitive antagonists [11,25,26,27,28].

**Figure 6:**
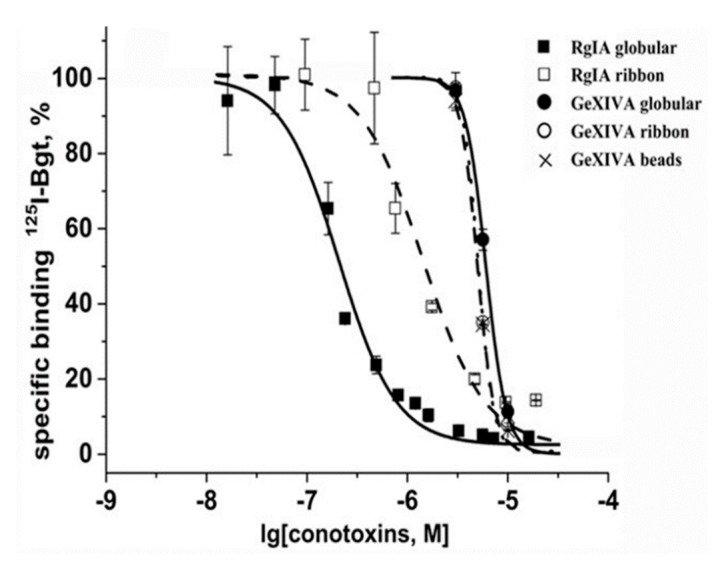
Inhibition of [^125^I]-labeled αBgt binding to *Aplysia californica* AChBP by two RgIA isomers (globular and ribbon) and three GeXIVA isomers (globular, ribbon and beads). The IC_50_ values were 194 ± 14 nM and 1.00 ± 0.08 μM for the globular and ribbon RgIA analogs, respectively; and around 2.9 μM for all GeXIVA isomers. Each point is the mean ± SEM value of two measurements for each concentration in two independent experiments.

In contrast to α9 LBD, which is not attaching to DE81 filters, AChBP binds with them with the same efficiency as with Ni^2+^-agarose beads. This feature allowed us to examine the binding potencies of RgIA and GeXIVA in competition with radioiodinated [^125^I]-αBgt for binding to *Aplysia californica* AChBP (Figure 6). The IC_50_ values of the conotoxins were in the range of 0.19–2.9 µM, with the most efficient being the RgIA globular isomer. These potencies are much weaker than nanomolar affinities of many other α-conotoxins towards *A. californica* AChBP [11,25,26,27,28], but we believe that this model protein could still be suitable for analyzing novel analogs of RgIA and GeXIVA.

As mentioned in the Introduction, α-conotoxins selective for the neuronal α9α10 nAChRs are promising potential analgesics. This group of marine toxins are usually peptides not longer than 16 amino-acid residues containing 2 disulfides, whose activity in most cases belongs to the isomers having globular conformation with the Cys1-Cys3 and Cys2-Cys4 disulfides (Figure 1) (see reviews [2,3,42]). Much hope was first placed on α-conotoxins Vc1.1 and RgIA having nanomolar affinity for rat α9α10 nAChRs, but later, they were found to be 100-fold weaker towards human α9α10 receptors. However, recently, a novel RgIA analog of high affinity and selectivity for human α9α10 has been designed and shown to be effective in a model of neuropathic pain [43,44]. 

A perspective for novel analgesics targeting α9α10 nAChRs appeared when, by the analysis of the conotoxin *Conus generalis* mRNA, an αO-conotoxin GeXIVA was discovered: it has a very high affinity for this receptor and shows much weaker action on all other tested nAChR subtypes [19]. It is longer (28 amino-acid residues) than usual α-conotoxins, and its all three synthesized isomers (globular, beads and ribbon) had comparable activity. According to electrophysiology measurements in *Xenopus* oocytes, this toxin binds to an allosteric, rather than the orthosteric, site, the latter being the classical site for agonists and competitive antagonists [19]. We decided to verify this conclusion by radioligand analysis, as we did recently with novel α-conotoxin PnIA analogs of high affinity for α7 nAChR by combining electrophysiology, calcium imaging, and αBgt competition, supplemented by the synthesis of radioactive α-conotoxin PnIA derivatives [33]. A promising material for such analysis of αO-conotoxin GeXIVA seemed to be the recombinant ligand-binding domain of the human nAChR α9 subunit (α9 LBD): the X-ray structures are known for this protein and for its complexes with the antagonists αBgt and methyllicaconitine [34]. 

Here, we found that three isomers of αO-conotoxin GeXIVA completely displaced radioiodinated αBgt from the α9 LBD with micromolar affinity, while globular α-conotoxin RgIA did the same with considerably lower potency (Figure 4). At first glance, these results suggest the interaction of both conotoxins with the same αBgt-binding (orthosteric) site on α9 LBD, but with low potency. What is the reason for a 1000-fold lower affinity for the α9 LBD, as compared to that for the heterologously expressed α9α10 nAChR? One explanation might be the presence of only one binding surface of the α9 subunit on the monomeric α9 LBD, while for high affinity binding of RgIA to the rat α9α10 nAChR, the most important was the contribution of the α10 subunit [14]. However, αBgt has retained its high affinity for the monomer α9 LBD, and this stimulated our further research on the nature of binding of GeXIVA and RgIA to this domain using their iodinated and radioiodinated derivatives. 

We found that radioiodinated derivatives of both conotoxins bind to Ni^2+^-agarose beads, required for attaching the His-tagged α9 LBD, almost with the same “visual” efficiency as to the α9 LBD domain itself (Figure 5C,D). The respective binding might be also the reason for such a big difference in the IC_50_ values between the α9α10 nAChR and α9 LBD for the noniodinated conotoxins.

The next step was the analysis of conotoxins RgIA and GeXIVA binding to another nAChR model, the *A. californica* AChBP. Because the majority of compounds which are capable of binding at the orthosteric sites of the muscle-type and homooligomeric nAChRs can also compete with radioiodinated αBgt for attaching to the AChBPs (see reviews [40,45]), it seemed appropriate to check the competition of conotoxins RgIA and GeXIVA with αBgt for attaching to this protein. The binding assay with *A. californica* AChBP does not require Ni^2+^-agarose resin, avoiding the problems faced with the His-tagged α9 LBD. It was shown that both conotoxins completely displaced radioiodinated αBgt at the micromolar concentrations by attaching at the orthosteric sites (Figure 6). As with most other α-conotoxins, in the case of RgIA, a higher affinity belongs to the globular isomer. 

## 3. Materials and Methods

Solid-phase synthesis of conotoxins. Synthesis of peptides was carried out using an automatic peptide synthesizer Myltisyntech Syro II. Preparative purification was carried out on a Gilson HPLC system (333/334 pump with 215 liquid handler and 155 UV detector, set at 210 and 280 nm). Peptides were eluted with a H_2_O-MeCN gradient with 0.1% trifluoroacetic acid (TFA). HPLC-MS analysis was performed for GeXIVA isomers using Thermo Finnigan LCQ Deca XP ion trap instrument with Thermo Accela UPLC system equipped with Waters Atlantis T3 column C_18_ (150 × 2 mm, 3um). Detection was achieved by UV-VIS DAD and full scan MS (ESI+, 150–2000 au). MALDI mass-spectrometry analysis for RgIA isomers was done using the time-of-flight mass spectrometer Ultraflex TOF/TOF (Bruker Daltonics, Germany). A solution of 2,5-dihydroxybenzoic acid (20 mg/ml, 50% acetonitrile in 0.1% TFA) was used as a matrix. The sample was applied to a MTP 384 target plate ground steel TF (Bruker Daltonics, Bremen, Germany) by a dried drop method. The samples were desorbed by irradiation with a nitrogen laser (wavelength 337 nm) operating at a frequency of 50 Hz. The analysis of the obtained mass spectrometric data was performed using the FlexAnalyses 3.0 software package (Bruker Daltonics, Bremen, Germany). 

A polystyrene resin with chlorotrityl chloride handle (2-CTC), Fmoc-protected amino acids and diisopropylcarbodiimide were from Iris Biotech (Marktredwitz, Germany). 4-Methylpiperidine was from Acros Organics (Belgium). Oxima pure was from EMD Chemicals. TFA was from Solvay S.A. (Brussels, Belgium). CLEAR-OX resin was from Peptides International. Acetonitrile was a gradient grade and received from Biosolve. All other reagents and solvents were purchased from a local manufacturer and used without additional purification. 

C-terminal amino acid was attached to the 2-CTC activated resin in the presence of Huenig’s base for 2 h. Peptide assembly was performed by Fmoc-methodology using diisopylcabodiimide activation with Oxima pure as nucleophilic additive. A 10-fold excess of amino acids was used within the 2 h condensation time. After the synthesis, the protected peptidyl-polymer was washed with diethyl ether, then dried and treated with TFA/DTT/H2O/TIS 150/4/3/0.5 (weight proportion) mixture. Next, 15 mL of the mixture was applied to 1 g of peptidyl-polymer during 2 h. Then, the solution was filtered out, the dry peptide was precipitated with 10-fold volume of diethyl ether, and remained at 4 °C for 8 h. The precipitated peptide was centrifuged, washed 3 times with diethyl ether, and then dried under vacuum. Crude peptide was purified by HPLC in a linear gradient of acetonitrile from 5 to 35% on a Silasorb-C_18_ (25 × 250 mm, 5 um) column, and was then lyophilized. 

In the case of α-conotoxin RgIA synthesis, a pure linear peptide was dissolved in 50 mM ammonium bicarbonate in water/acetonitrile 90:10 to a final concentration of 0.5 mg/mL. The resulting solution was stirred on air overnight, then acetonitrile was evaporated under vacuum, and residual solution was acidified by 1% v/v acetic acid and subjected to HPLC. After purification, the desired fractions were lyophilized and analyzed by MALDI-TOF mass spectrometry (see above). Thus, both isomers of RgIA were obtained. 

In the case of αO-conotoxin GeXIVA synthesis, linear peptide (with Trt- and Acm- protection of cysteines forming the first and second disulfide bonds, respectively) was dissolved in TFA to a final concentration of 2 mg/mL; then, equal volume of DMSO was added. Reaction mixture was allowed to stand for 24 h, then diluted tenfold by water and injected on a Silasorb-C_18_ (25 × 250 mm, 5 um) column. Elution was carried out in a linear gradient of acetonitrile from 5 to 35%; then, the desired fractions were lyophilized. Acm-deprotection and second disulfide bond formation were performed by treatment with fresh 50 mM solution of iodine in acetic acid. After exposition for 15 min, the peptide solution was diluted ten-fold by 10 mM solution of ascorbic acid and then lyophilized. Dark brown residue was dissolved in water and purified by chromatography on a Silasorb-C_18_ (25 × 250 mm, 5 um) column in a linear gradient of acetonitrile from 5 to 35%; it was then lyophilized and analyzed by electrospray HPLC-MS (see above). In this way, all three isomers of conotoxin GeXIVA were prepared. 

An alternative approach has been tested for the GeXIVA globular isomer: linear peptide was dissolved in a 1:1 mixture of 100 mM ammonium acetate buffer, pH 5.0, and acetonitrile to a final concentration of 4 mg/mL; this was then added to a 3 equivalents of preconditioned CLEAR-OX resin and shaken for 2 h. After that, the mixture was filtered, the resin was washed by an additional portion of buffer, and then, acetonitrile was evaporated from filtrates using rotavapor and the aqueous solution was directly injected on HPLC. Purified monocyclic peptide was dissolved in trifluoroacetic acid with 5% anisole to a final concentration of 2 mg/mL, and then cooled to 4 °C, and solution of 1 equivalent of thallium(III) trifluoroacetate was added. The reaction mixture was stirred overnight, then peptide was precipitated with ether and centrifuged. The crude folded peptide was purified by HPLC. The isolated sample was identical to the one obtained previously. All measured masses of peptides were in accordance with the calculated ones.

Preparation of [^127^I]- and [^125^I]-modified derivatives of α-conotoxin RgIA and αO-conotoxin GeXIVA and [^125^I]-α-bungarotoxin. Iodination of RgIA and GeXIVA conotoxins was carried out similarly to iodination of ImII (W10Y) α-conotoxin [22]. In 150 mM phosphate buffer (pH 7.0), 1 nmol of toxin, 0.7 nmol of Na [^127^I], and 7 nmol of chloramine T were incubated 10 min at 25°C. The reaction mixture was separated on a Reprosil-Pur C_18_ column (4 × 150 mm) (Dr. Maisch GmbH, Ammerbuch, Germany) with an acetonitrile gradient (with 0.1% TFA) from 5 to 35% in 30 min at a flow rate of 0.5 mL/min. Using this scheme, we prepared and collected for further work the mono-iodinated globular RgIA derivative (peak 3 in Figure 3A) and mixture of mono-iodinated beads GeXIVA derivatives (zone 3 in Figure 3B). Before radio-iodination, Na [^125^I] was isotopically diluted with Na [^127^I] to the chosen specific radioactivity in the range of 10-1000 Ci/mmol. After that, [^125^I]-labeled mono-iodinated RgIA and GeXIVA derivatives were obtained under the same conditions. Only mono-iodinated derivatives were used. 

The synthesis of the radioactive α Bgt was carried out essentially as described in [33]. For this study, a product with a specific radioactivity of 500 curies/mmol was obtained. For this, α Bgt (400 pmoles) dissolved in 20 μL of 125 mM sodium phosphate buffer, рН 7.5, was incubated for 10 min at room temperature with a mixture of 100 pmoles of Na [^125^I] and 300 pmoles of NaI and 10-fold molar 4 nmoles of chloramine T. After that, the reaction products were separated by ion-exchange HPLC in a 5 mM sodium-phosphate buffer, pH 7.5, in a gradient of 0.2 M NaCl (2–62% for 30 min) on a column TSKgel CM-5PW (75 × 7.5 mm) at a flow rate of 0.5 mL/min. Detection was carried out at 226 nm and the iodinated products were collected in 0.5 min-fractions. The aliquots of all fractions were counted on a Wizard 1470 Automatic Gamma Counter (Perkin Elmer, Shelton, CT, USA). Mono-[^125^I]iodinated α Bgt derivative (with approximate specific radioactivity of 500 Ci/mmol) was collected and kept at 4 °C in a 50 mM Tris-НС1 buffer, рН 7.5, containing 0.1 mg/ml BSA, for not more than 1 month. 

Analysis of competition of conotoxins with radioiodinated αBgt for binding to α9 LBD and A. californica AChBP. For competition binding assays, the heterologously-expressed *A. californica* AChBP (150 nm) was incubated in 50 μl of 20 mm Tris-HCl buffer, pH 8.0, containing 1 mg/mL BSA (binding buffer) for 90 min with various amounts of the conotoxins, followed by an additional 5-min incubation with 0.1–0.2 nm [^125^I]-labeled α-bungarotoxin (500 Ci/mmol). The *A. californica* AChBP samples were applied to two layers of DE-81 filters presoaked in phosphate-buffered saline containing 0.7 mg/mL BSA and washed (3 × 4 mL) with the same buffer. The samples were then washed (3 × 4 mL) with cold 20 mm Tris-HCl buffer, pH 8.0, containing 0.1 mg/mL BSA, and bound radioactivity was measured with a Wallac 1470 Wizard Gamma Counter (PerkinElmer, Waltham, MA, USA). 

For human α9 ECD saturation assay, 200 nmoles of this protein in its de-glycosylated form [34] were incubated in binding buffer with various [^125^I]-αBgt concentrations during 2 h at room temperature. Competition experiments were carried out with 100 nM α9 ECD and different amounts of the ligands, as described for *A. californica* AChBP. After incubation, 10 μL of Ni^2+^-NTA-agarose beads prewashed twice with binding buffer and diluted three times with the same buffer were added, and after additional 5 min incubation, suspensions were applied to glass GF/C filters, washed, and bound radioactivity was measured as described below. Nonspecific [^125^I]-αBgt binding was determined in the presence of 200-fold molar excess of α-cobratoxin.

Analysis of binding of radioiodinated RgIA and GeXIVA conotoxins with Ni^2+^-NTA-agarose. For this assay, human de-glycosylated α9 ECD (100 nM) was incubated in 50 μL of binding buffer (20 mM phosphate buffer, pH 7.0, containing 1 mg/mL BSA) with radioiodinated α-conotoxin RgIA (200 Ci/mmol) or αO-conotoxin GeXIVA (200 Ci/mmol) for 1 h. After incubation, 10 μL of Ni^2+^-NTA-agarose was added, and after an additional 5 min incubation, suspensions were filtered, washed, and bound radioactivity was measured as described above for *A. californica* AChBP. In the control binding assay, human α9 ECD was replaced by the same volume of binding buffer. Non-specific binding was determined in the presence of 200-fold molar excess of the respective unlabeled conotoxins.

Two-electrode voltage clamp analysis of interaction of conotoxins with the α9α10 nAChRs. Xenopus laevis frogs were fed twice a week and maintained according to supplier recommendations (https://www.enasco.com/page/xen_care). All the appropriate actions were taken to minimize discomfort to animals, and were carried out in accordance with the World Health Organization’s International Guiding Principles for Biomedical Research Involving Animals, under approval of IACUC (protocol number 251/2018 26 February 2018). 

Oocytes were removed from mature, anesthetized *Xenopus laevis* by dissecting the abdomen and removing necessary amount of ovarium. Stage V-VI oocytes were de-folliculated with 2 mg/mL collagenase Type I (Life Technologies, USA) at room temperature (21−24°C) for 2 h in in Ca^2+ ^ -free Barth’s solution composed of (in mM) 88 NaCl, 1.1 KCl, 2.4 NaHCO_3_, 0.8 MgSO_4_ and 15 HEPES-NaOH at pH 7.6. Oocytes were injected with 9.2 ng of rat or human nAChR α9 and α10 cRNA (in a ratio 1:1). Oocytes were incubated at 18 °C in Barth’s solution composed of (in mM) 88 NaCl, 1.1 KCl, 2.4 NaHCO_3_, 0.3 Ca(NO_3_)_2_, 0.4 CaCl_2_, 0.8 MgSO_4_ and 15 HEPES-10NaOH at pH 7.6, supplemented with 40 μg/mL gentamicin and 100 μg/mL ampicillin. Recordings were performed using turbo TEC-03X amplifier (Npi electronic, Tamm, Germany) and WinWCP recording software (University of Strathclyde, Glasgow, UK). The glass recording electrodes were filled with 3 M KCl, and the electrode resistance was 0.1–0.5 MΩ. Membrane potential was clamped at −60 mV. Oocytes were briefly washed with Ba^2+^ Ringer’s solution composed of (in mM) 115 NaCl, 2.5 KCl, 1.8 BaCl_2_, 10 HEPES at pH 7.2) followed by 3 applications of 10 μM of acetylcholine (ACh). Washout with Ba^2+^ Ringer’s was done for 5 min between ACh applications. Oocytes were pre-incubated with various concentrations of RgIA, [^127^I]-RgIA or GeXIVA, [^127^I]-GeXIVA for 5 min followed by its co-application with 10 μM ACh in case of α9α10 nAChR rat or human, respectively. Peak current amplitudes of ACh-induced responses were measured before (ACh alone) and after the pre-incubation of oocytes with peptides. The ratio between these two measurements was used to assess the activity of the tested compound.

## 4. Conclusions 

In view of the availability of the α9 LBD with the established X-ray structure [34], our main purpose was to confirm by radioligand analysis that αO-conotoxin GeXIVA indeed binds to an allosteric site as proposed from electrophysiological studies with the rat α9α10 nAChR [19]. However, this task was achieved neither for αO-conotoxin GeXIVA nor for α-conotoxin RgIA (which is believed to bind only to the orthosteric site), because these two toxins bound also to the Ni^2+^-NTA-agarose required for the attachment of the α9 LBD. Such binding was detected due to the synthesized radioiodinated derivatives of both conotoxins: according to electrophysiology on the α9α10 nAChR expressed in *Xenopus* oocytes, iodinated derivatives of both RgIA and GeXIVA had the same properties as the initial conotoxins. We believe that these radioidinated analogs will prove useful in further binding tests, which would eliminate need for the application of Ni^2+^-agarose resin.

Fortunately, during the performance of this work, additional information appeared on the biological activity of αO-conotoxin GeXIVA: it was found to be only about 5-fold less active towards human α9α10 nAChR [20]. In addition, some results were obtained in favor of the allosteric binding of αO-conotoxin GeXIVA to the α9α10 nAChR: namely, a number of mutations within the extracellular domain of the rat α9α10 nAChR did not affect the parameters of the currents inhibition by this toxin [20]. Still, we believe that clarifying the binding modes for conotoxins of such specificity—potential analgesics—deserves further research. In particular, an important question for drugs is their mode of action and selectivity for a particular nAChR subtype, and in this respect, the revealed orthosteric binding of RgIA and GeXIVA to AChBP, a general model of all nAChR subtypes, deserves attention.
